# Fatigue score as a promising complementary training monitoring tool: a pilot study in elite rugby sevens players

**DOI:** 10.5114/biolsport.2023.116011

**Published:** 2022-07-21

**Authors:** Emna Makni, Taieb Bouaziz, Karim Chamari, Raghad Tarwneh, Wassim Moalla, Mohamed Elloumi

**Affiliations:** 1 University of Sousse, Laboratory of Physiology and Physiopathology, Faculty of Medicine Ibn El Jazzar, Sousse, Tunisia; 2 Aspetar, Orthopaedic and Sports Medicine Hospital, FIFA Medical Centre of Excellence, Doha, Qatar; 3 Prince Sultan University, Health and Physical Education Department, Riyadh, Kingdom of Saudi Arabia; 4 UR15JS01. Education, Motricity, Sport and Health, High Institute of Sport and Physical Education, Sfax University, Tunisia

**Keywords:** Threshold of fatigue, Training load, Catecholamine, Glucocorticoid, Rugby sevens, Performances

## Abstract

The aim of this study was to compare physical and hormonal responses of seventeen elite rugby sevens players over a 6-week intense training block (IT) and a consecutive 2-week tapering period (TAP), using a fatigue cut-off score of 20 as a potential moderating variable. Training was monitored by daily training load (TL) and strain (TS) (using the session rating of perceived exertion [sRPE]) and also the weekly total score of fatigue (TSF; 8-item questionnaire tool). Testing and 24 h urinary cortisol (CL), cortisone (CN), adrenaline (AD) and noradrenalin (NAD) concentrations were also analysed before (T0) and after IT (T1) and after the TAP (T2). Players were assigned to group 1 with a TSF above 20 (G1 > 20, n = 9) and group 2 with a TSF below 20 (G2 < 20, n = 8) according to the French Society for Sports Medicine guidelines. TSF (effect size [ES] from 1.17 to 1.75), TL (ES from 0.81 to 1.06) and TS (ES from 1.23 to 1.40) were higher in G1 > 20 than in G2 < 20 over IT. Likewise, performance standards (ES from 1.58 to 2.61) and AD levels were lower (ES = 3.20), whereas CL and CL/CN ratio (ES from 1.60 to 3.47) were higher in G1 > 20 than in G2 < 20. After the TAP, TSF, TL and TS returned to baseline values for both groups, with an increase in performance standards and normalization in hormone levels. We suggest that a TSF greater than or equal to 20 could be considered as a fatigue threshold generating hormone disturbance and performance decrement, making it a potentially useful preventive and complementary training monitoring tool.

## INTRODUCTION

Rugby sevens has been an Olympic sport since 2016, resulting in a growing interest in the determinants of performance among players. Rugby sevens is considered as a physically demanding team sport requiring players to participate in frequent bouts of intense activities such as sprinting, physical collisions, and tackles interspersed by short bouts of low intensity activity such as walking and jogging [1]. Thus, performing in this team sport requires robust physical and psychological qualities, especially when performing at the highest level [2]. Managing training load and fatigue is considered an important component of the training process, especially in high training load sports such as rugby sevens; and optimizing training load will potentially result in an optimized performance [3–8]. Several parameters were previously employed to investigate potential physiological mechanisms underlying the progression towards excessive training (i.e., overreaching) that can result in detrimental performance. In this context, various physiological, haematological, biochemical, hormonal, and immunological parameters along with cardiovascular responses have been proposed as markers of these adaptations; however, nowadays there is no consensus on which measures are the most appropriate [3, 9–13]. Although the specific physiological factors underlying the progression towards overtraining syndrome remain unclear, research strongly highlights the importance of a psychological role in this context [14–16]. In fact, psychological factors, such as perceived training stress, anxiety and mood state, may also play a crucial role in high-level sports performance. Several practical tools have become available to team sports’ coaches to monitor the complex event of the stress/recovery balance in athletes and thus enable prevention of overreaching or overtraining [3, 7, 15, 17]. Self-reported measures (scales and questionnaires) are valid, practical, and simple tools for monitoring TL-induced psychological stress and fatigue [3, 7, 8, 11, 16–18]. The short questionnaire of fatigue, which stems from the large questionnaire of the French Society for Sports Medicine [17, 19, 20], allows an assessment of training stress, load, anxiety and strain in athletes [10, 18, 21] and has already been suggested as a helpful complement to the session rating of perceived exertion (sRPE) method for quantifying internal training load (TL) [3–7]. Interestingly, several studies have reported a significant relationship between the total score of fatigue (TSF) and the intensity of training variation as well as hormonal, performance and heart rate variability response-related training [3, 17, 20]. These findings suggest that both physiological and psychological parameters should be an integral part of the training process. Monitoring the latter sets of parameters could not only promote recovery, but also hopefully identify and prevent early signs of overtraining as previously suggested [14–16]. In this context, some authors [18, 19, 21] have proposed a TSF of 20 (arbitrary unit, AU) as a cut-off value for substantial fatigue in athletes. In this context, Atlaoui et al. [10] found that in response to an increase of training load, one swimmer showed a larger decrease in performance associated with a larger increase in TSF scores (from 19 AU to 28 AU) compared with the remaining swimmers of the study. Furthermore, Elloumi et al. [22] found that rugby players who presented higher TSF (mean 21 ± 3.5) exhibited larger significant hormone alterations than those who were less fatigued after an international competition. However, this cut-off value remains suggestive, for now relatively approximate, and to the best of our knowledge, no study has assessed its effectiveness. Therefore, the present pilot study aimed to compare physical and hormonal responses of seventeen elite rugby sevens players over a 6-week intense training block (IT) and a consecutive 2-week tapering period (TAP), using a fatigue cut-off score of 20 as a potential moderating variable.

## MATERIALS AND METHODS

Part of the dataset of this project has been published elsewhere [3]. The present study uses a different set of data with a subset of data that has been included in the previous study and another subset of data which is published here for the first time.

### Participants

Elite rugby players (rugby sevens, n = 17) from the Tunisian national team voluntarily participated in the study. They all regularly took part in national and international matches and their training schedule was as follows: 5 to 6 training weekly sessions (10–12 hours). Once the general preparation period was over, and up to the time of any international competitions, the players were used to training with the national team (2 training sessions daily, 3–4 hours) in addition to two rugby sevens games at the weekend, with only one day off weekly. The players participated in five high level international rugby sevens tournaments per year (organized by the International Rugby Board). The participants’ dietary intake was consistently administered, supervised and assessed by the national team’s nutritionist. All players were healthy, with no observed condition or treatment impeding or limiting their participation. Participants filled in a written informed consent form after having been informed of the study protocol, which has been approved by the institutional Ethical Committee. After having conducted our experiment, we assigned all the players to two groups according to the French Society for Sports Medicine guidelines and the suggestion by Chatard et al. [21]: group 1 with a TSF above 20: G1 > 20, and group 2 with a TSF below 20: G2 < 20.

### Training

The training programme consisted of 6-week IT (intense training block) and of 2-week TAP (tapering). Training sessions were focused on rebuilding physical conditioning of the players including an improvement of their aerobic capacity. Training included high-intensity interval runs, physical-technical circuits, and game-like activities with small groups and large spaces, with the intent that the intensity during the 4- to 6-minute series would be very high. The intensity, duration (volume) and frequency of sessions gradually increased during the period of IT and the duration and frequency declined steadily during the period of TAP. The players also performed sessions of speed and coordination training where speed, coordination and agility circuits were performed. Two specific-strength training sessions in the gymnasium (30–45 minutes) were performed before the field training to complement the physical training programme. For more details on the strength training programme, see Bouaziz et al. [3].

### Procedures and tests

The study was conducted during the preparation period for the rugby sevens World Cup held in 2013. Evaluation sessions (anthropometric performance measures) were conducted at the same time of the day at the National Center of Medicine and Science in Sports, Tunis, Tunisia (temperature: 18 ± 2°C, and relative humidity 44 ± 8%) where players were assessed at three time points:

1. At (T0): previously to the training programme,
2. At (T1): after the 6 weeks of IT training (intense training block),
3. At (T2): after a 2-week TAP (tapering) that came immediately after IT.

**FIG. 1:**
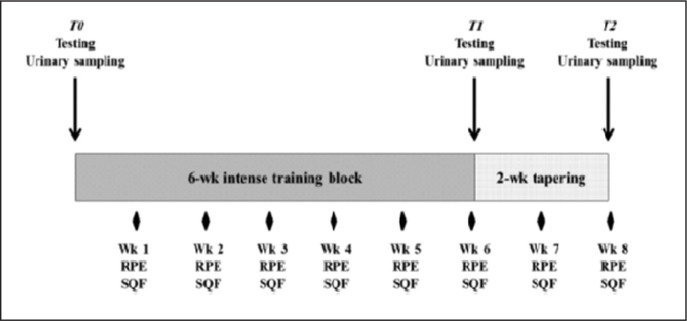
Schematic overview of the study design. Note: sRPE: session-rating of perceived excretion. SQF: short questionnaire of fatigue.

The physical assessments were part of the players’ fitness assessments according to sevens World Cup rules (Figure 1). Body mass, height, and percent body fat (% BF) were assessed using calibrated tools [23].

### Physical tests

The players performed the following physical tests: 30-m sprints (sprint), five-jump test (leg explosiveness), Illinois agility run (agility), Australian lactic test (mostly anaerobic test), one maximum repetition (1-RM) of bench press and half squat (strength), and the Yo-Yo intermittent recovery test level 2 (Yo-Yo IRT2 – endurance). For more details see Bouaziz et al. [3].

### Training load monitoring

Training load, monotony and strain for each participant were collected approximately 30 min after the end of each session and were calculated according to the sRPE method [4]. The weekly training strain was then calculated as the product of weekly training load and monotony. The mean training load and strain were also calculated for the 6-week IT and the 2-week TAP. The training load, monotony and strain are expressed in arbitrary units (AU).

### Short questionnaire of fatigue

Chatard et al. [21] described the short questionnaire of fatigue which consists of eight questions focusing on: the perception of training, leg pain, concentration, efficacy, sleep, infection, anxiety, irritability, and general stress (questions being assessed on a 7-point scale: from 1 point (not at all) to 7 points (very much)). The summed 8 responses allowed calculation of the TSF (total score of fatigue). The TSF, TL (training load) and TS (training strain) during the IT (6-week) and TAP (2-week) were independently averaged for each group, in order to assess the possible association between these variables and (i) the physical performance and (ii) the 24 h urinary hormonal excretion changes during these two periods.

### Urine samples

Each week during the protocol, urinary samples were collected from all the participants over a 24 h resting period [3]. Catecholamine and urinary glucocorticoid concentrations were assessed [high performance liquid chromatography (HPLC)] [24, 25]. Intra- and inter-assay coefficients of variation were < 3% for catecholamines and < 1% for glucocorticoids (both were expressed in µg · mg^-1^ of creatinine per 24 h; furthermore they were determined in duplicate).

### Statistical analysis

Data are presented as mean ± SD and statistical analyses were performed using the SPSS package (SPSS Inc., Chicago, IL, version. 16.0). After checking the normality of data distribution using the Shapiro-Wilk test, an independent t-test was performed to determine significant differences between groups in baseline values. To assess and compare physical and hormonal responses in sevens rugby players according to low and high TSF, a two (G1 > 20 and G2 < 20) × 3 (time: pre-training, post-6-week IT and post-2-week TAP) analysis of variance (ANOVA) with time as the repeated within-participant factor was used. Bonferroni post-hoc testing was then performed to identify any differences following a significant group × time interaction effect. For each variable (test and hormone level), partial eta-squared values (ηp2) were used for effect size calculation (with ηp2 up to 0.059 = small; between 0.059 and 0.138 = medium and greater than 0.138 = large) [26]. Additionally, between-group standardized mean differences or effect sizes (ES) of pre-training, pre-training to post-6-week IT and pre-training to post-2-week TAP in performance and hormone changes were calculated using Cohen’s *d* and corrected by Hedge’s *g* as our sample size was small (< 20) to avoid a biased estimation of the population effect size provided by Cohen’s *d*. According to Cohen, ES can be classified as small (0 ≤ d ≤ 0.49), medium (0.50 ≤ d ≤ 0.79), and large (d ≥ 0.80) [27]. The level of significance was set at p ≤ 0.05.

## RESULTS

All participants attended all training sessions with no test or training-related reported injuries. Table 1 shows anthropometrics and physical performances and Table 2 shows urinary hormone concentration results.

**TABLE 1:** Anthropometric and physical performance data over the 8-wk training period in GTSF > 20 and GTSF < 20.

	GTSF > 20 (n = 9)			GTSF < 20 (n = 8)			η_p_^2^ (group)/*p* value	η_p_^2^ (Time)/*p* value	η_p_^2^ (time × group)/*p* value
	T_0_	T_1_	T_2_	T_0_	T_1_	T_2_			
Age (year)	24.8 ± 3.8	-	-	22.7 ± 1.3	-	-			
Height (cm)	1.82 ± 0.07	-	-	1.84 ± 0.06	-	-			
Body mass (kg)	86.8 ± 8.4	85.0 ± 7.9^*^	85.1 ± 7.9^*^	88.1 ± 5.8	86.6 ± 6.2^*^	86.7 ± 6.1^*^	0.01/0.70	0.72/0.000	0.01/0.85
Fat mass (%)	14.2 ± 3.0	12.2 ± 2.6^*^	12.2 ± 2.7^*^	11.6 ± 1.4	10.0 ± 1.4^*^	9.9 ± 1.5^*^	0.23/0.06	0.79/0.000	0.05/0.52
Lean mass (kg)	74.4 ± 6.9	74.6 ± 7.0	74.7 ± 7.1	77.8 ± 4.8	77.9 ± 4.8	78.0 ± 4.7	0.08/0.30	0.32/0.004	0.009/0.88
10-m sprint (s)	1.82 ± 0.10	1.88 ± 0.07^**^	1.79 ± 0.09^*^	1.82 ± 0.04	1.84 ± 0.05^*^	1.80 ± 0.04^*^	0.003/0.85	0.56/0.000	0.16/0.08
20-m sprint (s)	3.13 ± 0.08	3.17 ± 0.09^*^	3.07 ± 0.09^*^	3.12 ± 0.06	3.14 ± 0.07^*^	3.09 ± 0.06^*^	0.004/0.83	0.65/0.000	0.23/0.03
30-m sprint (s)	4.28 ± 0.17	4.41 ± 0.15^**^	4.28 ± 0.14	4.31 ± 0.10	4.36 ± 0.09^*^	4.29 ± 0.09^*^	0.000/0.98	0.41/0.001	0.10/0.23
AGT (s)	16.78 ± 0.37	17.25 ± 0.34^*^	16.32 ± 0.50^*^	16.65 ± 0.31	16.95 ± 0.31^*^	16.37 ± 0.19^*^	0.01/0.72	0.63/0.000	0.09/0.26
FJT (m)	11.8 ± 0.5	11.4 ± 0.4^**^	12.8 ± 0.6^*^	11.4 ± 0.8	11.2 ± 0.8^*§^	12.5 ± 1.3^*^ 5	0.1/0.24	0.62/0.000	0.004/0.95
LT (m)	718.9 ± 42.5	709.9 ± 41.8	736.3 ± 35.8^*^	696.4 ± 24.7	692.4 ± 23.3	715.1 ± 16.4^*^	0.1/0.24	0.73/0.000	0.03/0.68
Yo-YoIRT2 (m)	1728.9 ± 394.9	1604.4 ± 395.7^**^	1902.2 ± 423.8^*^	1731.4 ± 199.6	1651.4 ± 184.3^*§^	1954.3 ± 188.2^**^	0.003/0.84	0.92/0.000	0.08/0.31
1RM SQT (kg)	166.4 ± 19.9	152.6 ± 18.3^**^	170.4 ± 18.6^*^	165.6 ± 10.7	158.9 ± 12.8^*^55	170.3 ± 10.2^*^	0.003/0.87	0.92/0.000	0.31/0.006
1RM BP (kg)	116.2 ± 8.8	106.3 ± 10.7^**^	118.6 ± 9.3^*^	112.9 ± 11.2	108.6 ± 10.4^*§§^	117.4 ± 12^**§§^	0.002/0.87	0.87/0.000	0.44/0.000

Note: GTSF > 20: group of players with total score of fatigue above 20; GTSF < 20: group of players with total score of fatigue below 20; AGT: agility test; FJT: five jump test; LT: Lactic test; YoYoIRT2: YoYo intermittent recovery test level 2; 1RM: maximum repetition; SQT: squat; BP: bench press. * Statistical difference within group from T_0_;

* *p* < 0.05,

** *p* < 0.01. § Statistical difference from GTSF > 20 at the same time of the training program;

§ P < 0.05,

§§ P < 0.01.

**TABLE 2:** Changes in urinary hormones and their ratios over 8-week training program in GTSF > 20 and GTSF < 20.

	GTSF > 20 (n = 9)			GTSF < 20 (n = 7)			η_p_^2^ (group)/*p* value	η_p_^2^ (Time)/*p* value	η_p_^2^ (time × group)/*p* value
	T_0_	T_1_	T_2_	T_0_	T_1_	T_2_			
CL de 24 h (μg · mg^-1^ of creatinin)	17.4 ± 1.4	24.7 ± 2.6^**^	17.9 ± 1.4	16.8 ± 0.6	21.9 ± 1.0^**§§^	17.0 ± 0.5	0.25/0.051	0.95/0.000	0.35/0.003
CN de 24 h (μg · mg^-1^ of creatinin)	22.5 ± 1.9	24.7 ± 2.4^*^	22.9 ± 1.8	21.7 ± 1.1	25.2 ± 1.7^**§^	21.9 ± 1.0	0.01/0.70	0.88/0.000	0.30/0.007
CL/CN ratio	0.78 ± 0.05	1.00 ± 0.10^**^	0.78 ± 0.06	0.77 ± 0.03	0.87 ± 0.06^*^55	0.78 ± 0.02	0.17/0.12	0.89/0.000	0.55/0.000
AD de 24 h (μg · mg^-1^ of creatinin)	10.4 ± 0.5	6.2 ± 1.3^**^	12.3 ± 0.7^*^	9.4 ± 0.8	8.1 ± 0.9^*§§^	10.0 ± 1.5	0.03/0.56	0.85/0.000	0.47/0.003
NAD de 24 h (μg · mg^-1^ of creatinin)	24.4 ± 1.2	18.5 ± 1.0^**^	26.6 ± 1.1^*^	25.0 ± 1.1	18.5 ± 0.8^**^	27.6 ± 1.2^*§^	0.09/0.70	0.98/0.000	0.13/0.14
AD/NAD ratio	0.43 ± 0.02	0.34 ± 0.10^**^	0.46 ± 0.07^*^	0.38 ± 0.03	0.44 ± 0.05^**§§^	0.40 ± 0.05^*^	0.08/0.28	0.35/0.003	0.56/0.000

Note: GTSF > 20: group of players with total score of fatigue above 20; GTSF < 20: group of players with total score of fatigue below 20; CL: cortisol; CN: cortisone; AD: adrenaline; NAD: noradrenaline. * Statistical difference within group from T0;

* *p* < 0.05,

** *p* < 0.01. § Statistical difference from GTSF > 20 at the same time of the training program;

§ *P* < 0.05,

§§ *P* < 0.01.

### Pre-training data

Pre-training data showed no statistically significant differences for anthropometric, physical tests, and urinary hormone variables between groups except for AD (ES = 1.68) and AD/NAD ratio (ES = 2.02).

### TSF, TL and TS

**FIG. 2:**
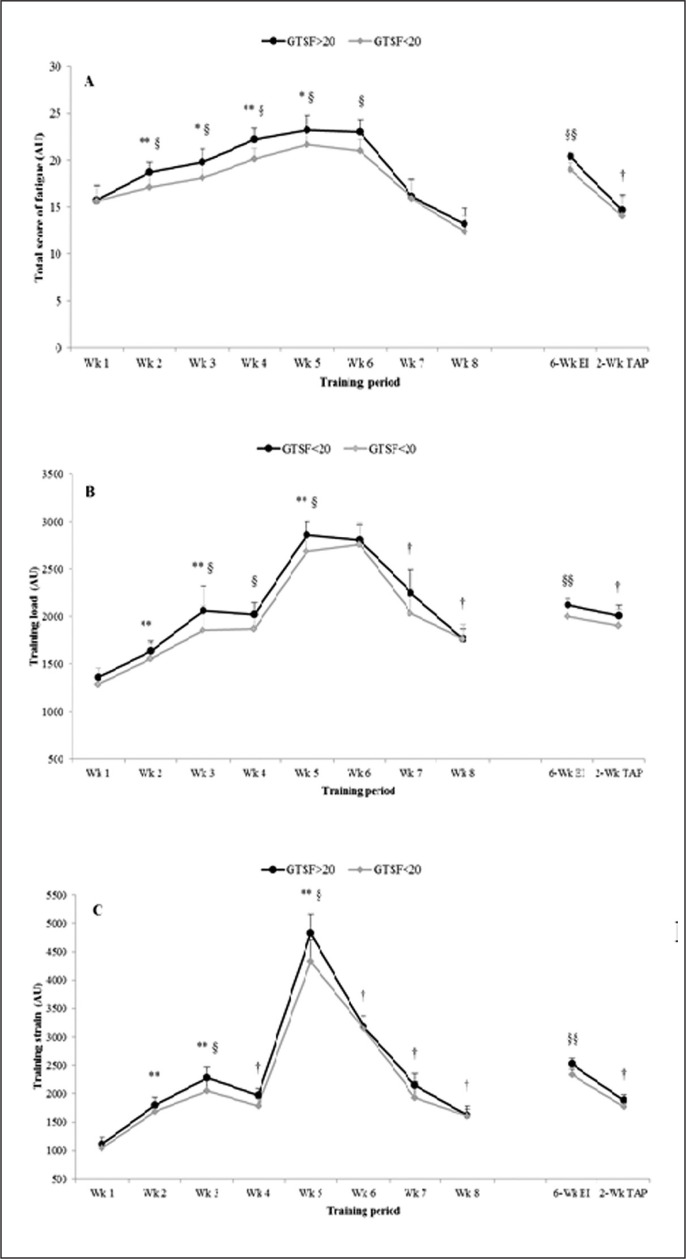
Total score of fatigue (A), training load (B) and training strain (C) recorded over the 8-week training program in GTSF > 20 and GTSF < 20. Note: *: higher than the precedent value, *p < 0.05, **p < 0.01; †: lower than the precedent value, †*p* < 0.05, ††*p* < 0.01; §: higher than the GTSF < 20, §p < 0.05, §§p < 0.01.

The TSF, TL and TS are reported in Figures 2A, 2B and 2C, respectively. TSF increased until reaching a peak value over the 5^th^ week during the 6-week IT period in both groups. This increase was associated with simultaneously increased values of TL and TS with the highest score recorded during the 5^th^ week. Significant interactions were found (training × group) for TSF, TL and TS (F(1,14) = 30.48, ηp2 = 0.69; F(1,14) = 9.17, ηp2 = 0.40; F(1,14) = 11.29, ηp2 = 0.45, respectively). Post-hoc analysis revealed that the increase in TSF was significantly larger in G1 > 20 compared to G2 < 20 at the 2^nd^, 3^rd^, 4^th^, 5^th^ and 6^th^ week of the IT period (ES from 1.17 to 1.75; p < 0.01). Similarly, the increase in TL and TS was significantly larger in G1 > 20 compared to G2 < 20 at the 3^rd^ (ES = 1.23 and ES = 0.81, respectively) and the 5^th^ (ES = 1.06 and ES = 1.42, respectively) week of the IT period. Conversely, all the parameters decreased significantly during the 2-week TAP. TSF, TL and TS decreased significantly during the TAP in both groups (p < 0.01). No significant difference in TSF, TL and TS values was recorded between groups during TAP.

### Testing performances

Significant interactions were found (training × group) for 20-m sprint, 1RM squat (SQT) and 1RM bench press (BP) performances (F(1,14) = 4.15, ηp2 = 0.23; F(1,14) = 6.28, ηp2 = 0.31 and F(1,14) = 10.87, ηp2 = 0.44, respectively). Indeed, after the IT period (T1), all performances significantly decreased in G1 > 20 and G2 < 20. Conversely, the TAP (T2) resulted in a significant increase in all testing performances in both groups (Figure 2A). At T1, the decreases in performances of the five-jump test (FJT) (Δ-3.5% vs Δ-1.2%; p < 0.05, ES = -1.58), Yo-YoIRT2 (Δ-7.5% vs Δ-4.6%; p < 0.05, ES = -1.63), 1RM SQT (Δ-8.4% vs Δ-4.1%; p < 0.01, ES = -2.19) and 1RM BP (Δ-8.6% vs Δ-3.8%; p < 0.01, ES = -2.61) were significantly larger in G1 > 20 compared to G2 < 20. At T2, the increase in 1RM BP was significantly larger in G2 < 20 compared to G1 > 20 (Δ+4% vs Δ+2%; p < 0.05, ES = 1.55).

### Urinary hormonal changes

**FIG. 3:**
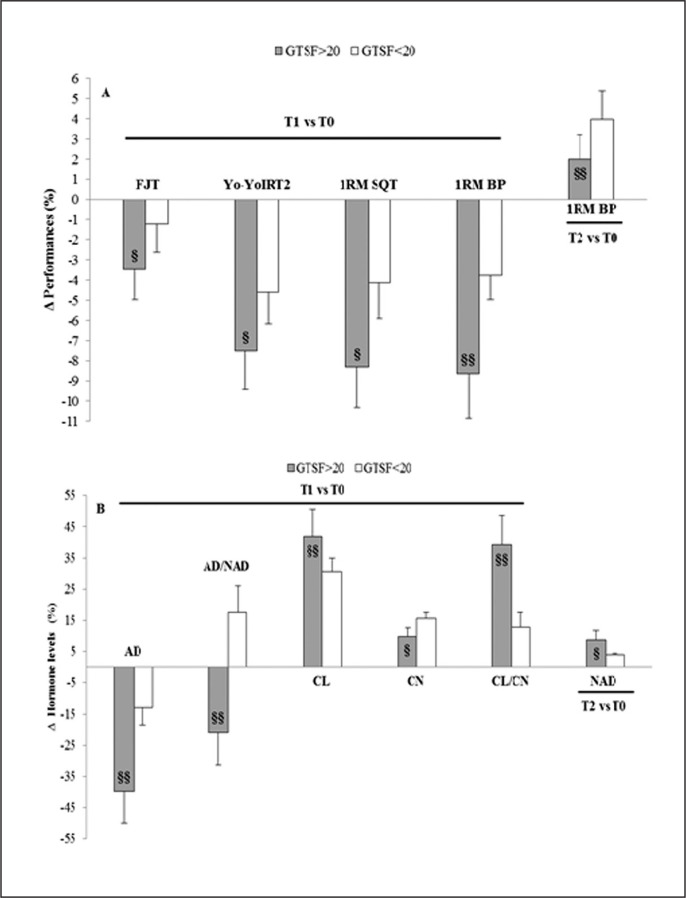
Changes in testing performances (A) and urinary hormonal levels (B) over the two periods of the training program in GTSF > 20 and GTSF < 20. Note: GTSF > 20: group of player with a total score of fatigue above 20; GTSF < 20: group of players with a total score of fatigue below 20; FJT: five jump test; YoYoIRT2: YoYo intermittent recovery test level 2; 1RM: one maximum repetition; SQT: squat; BP: bench press, AD: adrenaline; NAD: noradrenaline; AD/NAD: Adrenaline/Noradrenaline ratio; CL: cortisol; CN: cortisone; CL/CN: cortisol/cortisone ratio. §: different from the GTSF < 20; §p < 0.05, §§p < 0.01.

Significant interactions were found (training × group) for CL, CN, CN/CL ratio, AD and AD/NAD ratio values (F(1,14) = 7.46, ηp2 = 0.35; F(1,14) = 5.95, ηp2 = 0.30; F(1,14) = 16.90, ηp2 = 0.55; F(1,14) = 12.36, ηp2 = 0.47 and F(1,14) = 17.73, ηp2 = 0.56, respectively). After the 6-week IT, CL, CN and CL/CN ratio increased significantly while AD and NAD levels decreased significantly in both groups. Compared to T0, CL, CN and CL/CN ratio in T2 returned to baseline values whereas AD and NAD remained significantly higher, especially in G1 > 20 (Figure 2B). At T1, the increase in CL (Δ+41.8% vs Δ+30.5%; ES = 1.60) and CL/CN ratio (Δ+39.2% vs Δ+12.8%; ES = 3.47) and the decrease in AD (Δ-39.8% vs Δ-12.9%; ES = -3.20) were significantly larger in G1 > 20 compared to G2 < 20. AD/NAD ratio increased in G2 < 20 and decreased in G1 > 20 (Δ+17.7% vs Δ-21.0%; ES = 4.05). In addition, CN level was significantly higher in G2 < 20 compared to G1 > 20 (Δ+15.7% vs Δ+9.9%; ES = 2.36). At T2, the increase in NAD level was significantly larger in G1 > 20 compared to G1 < 20 (Δ+8.8% vs Δ+4.0%; ES = 2.17) (Figure 3).

## DISCUSSION

The aim of the present study was to examine the effectiveness of a cut-off level of fatigue score of 20 in elite rugby seven players during an 8-week training camp, including a 6-week intense training block (IT) and 2-week tapering (TAP). Accordingly, we compared physical and hormonal responses of two groups of players using this score as a potential moderator variable (G1 > 20 and G2 < 20). The main findings indicated that a high training load programme generated a significantly large increase (with large effect size) in TSF, TL and TS in G1 > 20 compared to G2 < 20, which was associated with significantly greater alteration in hormone levels and physical performances in the G1 > 20. The data also demonstrated that following the 2 weeks of TAP all variables returned to baseline values in both groups while the NAD level remained higher in G1 > 20 compared to G2 < 20. This result is concomitant with similar improvement in both groups of physical performance, except the 1RM BP, which became higher in G2 < 20.

It was pointed out that managing athlete stress and fatigue is crucial in monitoring athlete loads. This is particularly important in terms of the measures which may offer insights into whether the athlete is adapting positively or negatively to the training and competition stress. The present data corroborate several previous studies that investigated a “global” sense of the relationship between the perceptual fatigue-related training and physiological, hormonal, neuromuscular and cardiovascular parameters [11, 17, 20]. Indeed, a deeper approach has been recommended by the French Society for Sport Medicine to detect and to prevent an early state of fatigue, when they suggested a TSF of 20 as the threshold of this fatigue state [19]. In this context, several authors have adopted the threshold score of 20 since it is considered as an alarm signal of a state of fatigue or non-functional overtraining [18, 21, 22]. However, further confirmation studies are needed in this regard.

The high TL and TS observed during the initial 6-week IT is concomitant with the larger increase of urinary CL and CN levels and CL/CN ratio and conversely with the larger decrease of urinary AD and NAD levels in G1 > 20 compared to G2 < 20. Previous studies examining the effect of training programmes on salivary or plasma catabolic hormones have presented discrepant results in team-sport athletes [11, 20, 28, 29]. Kraemer et al. [30], Coutts et al. [28, 29] and Campos et al. [11] reported significantly higher resting saliva or plasma cortisol levels with performance impairments in soccer and rugby players as well as individual athletes. Conversely, Elloumi et al. [17] reported decreased performance in rugby league players over a 14-week training programme despite unaltered resting saliva cortisol levels. The discrepant results could be explained by several factors such as sampling methods, circadian rhythm, and cortisol metabolism. It is well accepted that cortisol secretion follows a circadian rhythm with significant fluctuation of its plasma or salivary concentrations between awakening and the evening nadir. In fact, the major benefit of 24 h urinary collection is that the measure of the urinary hormone excretion represents both a good reflection of hormonal secretion under the time of sampling and a non-stressful measurement [31, 32].

The present study also highlighted a significant increase in CL and CN levels as well as CL/CN ratios between T0 and T1. Likewise, TL, TS and TSF showed higher values associated with a significantly larger decline in physical performance (large effect) in G1 > 20 compared to G2 < 20. When considering previous studies examining CL, CN and CL/CN ratio changes over intensified training periods [9, 10]; it appears that such higher hormone levels may be explained in part by a higher responsiveness of the hypothalamopituitary-adrenal axis to physiological adaptation of the neuroendocrine system to chronic exercise demands, without ruling out a potential modification of the clearance of these hormones. Interestingly, consistently with the findings of Atlaoui et al. [9] and Rouveix et al. [13], the increased CL/CN ratio as well as CL and CN levels was associated with increased TSF, TL and TS and decreased physical performances. In addition, Atlaoui et al. [9] reported that the CL and CN concentrations of one swimmer, who had high fatigue scores, were higher than those of the other less fatigued swimmers. In line with this, Elloumi et al. [22] observed a decrease of somatomedin axis hormones (anabolic effect) after an international rugby match in more fatigued players (TSF; 21.0 ± 3.5). The latter authors suggested that low levels of this hormone are linked to a state of fatigue. The association of increased CL and CN levels as well as CL/CN ratio with decreased performance standards at T1 is in agreement with previous conclusions indicating catabolic state-related training [3, 9, 13]. This catabolic state was more pronounced in G1 > 20 compared to G2 < 20.

The training programme also induced lowered urinary AD and NAD levels as well as AD/NAD ratio compared to pre-training values in both groups (especially in G1 > 20). The decreased catecholamine with IT is in agreement with previous research on swimmers, tennis players and rugby sevens players [3, 9, 12, 13] but inconsistent with those reported in 18 semi-professional rugby league players [28, 29]. A possible reason for the differences between Coutts’s findings and the present results is that the training load and strain increased steeply during the last two weeks of IT in our study, whereas in the studies by Coutts et al. [28, 29], the athletes were intentionally overloaded with a progressive increase of training load and strain. It has been pointed out that repeated exposure to stressful conditions related to exercise training, such as the rugby training performed in the present study, is frequent but not always accompanied by a reduction of stress-induced catecholamine secretion [9, 12, 13]. Because NAD is mostly affected by physical stress while AD rather more by mental stress [33], we believe that the magnitude of decrease of AD in G1 > 20 is due to mental stress related to training. Accordingly, the decrease of AD/NAD ratio reported in G1 > 20 after the initial 6-week IT can partly be explained by the reduction of sympatho-adrenomedullary activity with intensified training [10, 12]. However, the phenomenon of homeostasis disturbance was transient since the study’s participants showed an ability to improve their performance standards following a short-term regeneration period (i.e. 2-week TAP). Concurrently with these performance’s positive responses, CL/CN and AD/NAD ratios returned to their baseline values. It has been pointed out that exercise training sessions cause transient changes in physiological function that, when repeated over time, predispose the exercising organism to beneficial adaptations [34]. The short-term step taper completed in this study allowed for overcompensation in the majority of the measured physical performances, along with a return to a homeostasis environment especially in G2 < 20. Indeed, another salient finding of the present study was that G1 > 20 did not exhibit complete homeostasis compared with G2 < 20, resulting in a higher value of AD and a smaller improvement in 1MRBP after TAP. Overall, these data showed that 2-week tapering, suggested as the most efficient strategy to maximize performance gains [35], generates physiological and psychological complete recovery. These results are also in agreement with previous studies in team-sport athletes [3, 28, 29, 36]. Therefore, we suggest that a value of 20 units for TSF could be considered as a cut-off level above which performance could be decreased, potentially resulting in overreaching if the training load is not adjusted. To confirm or to complement these results, further research is needed in larger cohorts and/or other team sports. Importantly, the individual variations in the TSF should be examined in relation to changes in performance and biological markers throughout an extensive follow-up where fatigue occurs. The present study has some limitations that should be recognized. Despite the use of TSF and sRPE procedures which have been used to quantify training load and fatigue in high level rugby sevens players [3], we did not assess other variables such as heart rate, self-reporting of stress, fatigue, muscle soreness and quality of sleep in addition to other biochemical/hormonal and immunological variables, which are all considered as internal training load indices that could have increased the value of our study. These should be considered by future investigations in the field.

## CONCLUSIONS

The findings support the suitability of the TSF in identifying rugby sevens players with high training-related levels of fatigue that are associated with negative physiological responses. A cut-off threshold higher than or equal to 20 appears to be an alarming signal of high fatigue condition and potentially an overload to be considered for training adjustments. Two weeks of tapering allowed the homeostasis state to revert back to pre-training levels. This suggests that this level of fatigue is easily detectable and still rapidly revertible. Further studies are required to either reinforce the effectiveness of this score value, or to adjust it according to high-level athletes’ adaptations.

## References

[1] Ross A, Gill N, Cronin J. Match analysis and player characteristics in rugby sevens. Sports Med. 2014; 44(3):357–367. 24234931 10.1007/s40279-013-0123-0

[2] Kruyt N, Grobbelaar H. Psychological demands of International Rugby Sevens and well-being needs of elite South African players. Front Psychol. 2019:29; 10:676. doi: 10.3389/fpsyg.2019.00676. eCollection 2019. PMC6450168 30984080

[3] Bouaziz T, Makni E, Passelergue P, Tabka Z, Lac G, Moalla W, et al. Multifactorial monitoring of training load in elite rugby sevens players: cortisol/cortisone ratio as a valid tool of training load monitoring. Biol Sport. 2016; 33:231–239. 27601777 10.5604/20831862.1201812 PMC4993138

[4] Foster C, Florhaug JA, Franklin J, Gottschall L, Hrovatin LA, Parker S, et al. A new approach to monitoring exercise training. J Strength Cond Res. 2001; 15:109–115. 11708692

[5] Lukonaitienė I, Conte D, Paulauskas H, Pliauga V, Kreivytė R, Stanislovaitienė J, et al. Investigation of readiness and perceived workload in junior female basketball players during a congested match schedule. Biol Sport. 2021; 38(3):341–349. 34475617 10.5114/biolsport.2021.99702 PMC8329967

[6] Marynowicz J, Lango M, Horna D, Kikut K, Andrzejewski M. Predicting ratings of perceived exertion in youth soccer using decision tree models. Biol Sport. 2022; 39(2):245–252. 35309546 10.5114/biolsport.2022.103723 PMC8919883

[7] Moalla W, Fessi MS, Farhat F, Nouira S, Wong DP, Dupont G. Relationship between daily training load and psychometric status of professional soccer players. Res Sports Med. 2016; 24:387–394. 27712094 10.1080/15438627.2016.1239579

[8] Oliveira R, Brito J, Martins A, Mendes B, Calvete F, Carriço S, et al. In-season training load quantification of one-, two- and three-game week schedules in a top European professional soccer team. Physiol Behav. 2019; 201:146–156. 30529511 10.1016/j.physbeh.2018.11.036

[9] Atlaoui D, Duclos M, Gouarne C, Lacoste L, Barale F, Chatard JC. The 24-h urinary cortisol/cortisone ratio for monitoring training in elite swimmers. Med Sci Sports Exerc. 2004; 36:218–224. 14767243 10.1249/01.MSS.0000113481.03944.06

[10] Atlaoui D, Duclos M, Gouarne C, Lacoste L, Barale F, Chatard JC. 24-hr urinary catecholamine excretion, training and performance in elite swimmers. Int J Sports Med. 2006; 27:314–21. 16572375 10.1055/s-2005-865669

[11] Campos F, Molina Correa JC, Canevari VCM, Branco BHM, Andreato LV, de Paula Ramos S. Monitoring Internal Training Load, Stress-Recovery Responses, and Immune-Endocrine Parameters in Brazilian Jiu-Jitsu Training. J Strength Cond Res. 2020; 16. doi: 10.1519/JSC.0000000000003507. 31972820

[12] Filaire E, Rouveix M, Duclos M. Training and 24-hr urinary catecholamine excretion. Int J Sports Med. 2009; 30:33–39. 18651370 10.1055/s-2008-1038758

[13] Rouveix M, Duclos M, Gouarne C, Beauvieux MC, Filaire E. The 24 h urinary cortisol/cortisone ratio and epinephrine/norepinephrine ratio for monitoring training in young female tennis players. Int J Sports Med. 2006;27:856–63. 16586341 10.1055/s-2006-923778

[14] Kellmann M. Preventing overtraining in athletes in high-intensity sports and stress/recovery monitoring. Scand J Med Sci Sports. 2010; Suppl 2:95–102. 10.1111/j.1600-0838.2010.01192.x 20840567

[15] Nicolas M, Vacher P, Martinent G, Mourot L. Monitoring stress and recovery states: Structural and external stages of the short version of the RESTQ sport in elite swimmers before championships. J Sport Health Sci. 2019; 8:77–88. 30719387 10.1016/j.jshs.2016.03.007 PMC6349564

[16] Saw AE, Main LC, Gastin PB. Monitoring the athlete training response: Subjective self-reported measures trump commonly used objective measures: A systematic review. Br J Sports Med. 2016; 50:281–291. 26423706 10.1136/bjsports-2015-094758 PMC4789708

[17] Elloumi M, Ben Ounis O, Tabka Z, Van Praagh E, Michaux O, Lac G. Psychoendocrine and physical performance responses in male Tunisian rugby players during an international competitive season. Aggress Behav. 2008; 34:623–632. 18626966 10.1002/ab.20276

[18] Chatard JC, Stewart AM. Training load and performance in swimming,” in World Book of Swimming: From Science to Performance, Editors: Seifert L, Chollet D, Mujika I. Nova Science Publishers, Inc 2011:359–373.

[19] Maso F, Lac G, Brun JF. Analysis and interpretation of SFMS questionnaire for the detection of early signs of overtraining:a multicentric study. Sci Sport. 2005; 20:12–20.

[20] Parrado E, Cervantes J, Pintanel M, Rodas G, Capdevila L. Perceived tiredness and heart rate variability in relation to overload during a field hockey World Cup. Percept Mot Skills. 2010; 110:699–713. 20681325 10.2466/PMS.110.3.699-713

[21] Chatard JC, Atlaoui D, Pichot V, Gouarné C, Duclos M, Guézennec YC. Training followed by questionnaire fatigue, hormones and heart rate variability measurements. Sci Sport. 2003; 18:302–304.

[22] Elloumi M, El Elj N, Zaouali M, Maso F, Filaire E, Tabka Z, et al. IGFBP-3, a sensitive marker of physical training and overtraining. Br J Sports Med. 2005; 39:604–610. 16118296 10.1136/bjsm.2004.014183 PMC1725308

[23] Durnin JV, Womersley J. Body fat assessed from total body density and its estimation from skinfold thickness: measurements on 481 men and women aged from 16 to 72 years. Br J Nutr. 1974; 32:77–97. 4843734 10.1079/bjn19740060

[24] Hay M, Mormède P. Improved determination of urinary cortisol and cortisone, or corticosterone and 11-dehydrocorticosterone by high-performance liquid chromatography with ultraviolet absorbance detection. J Chromatogr B Biomed Sci Appl. 1997a; 702:33–39. 9449553 10.1016/s0378-4347(97)00361-7

[25] Hay M, Mormède P. Determination of catecholamines and methoxy-catecholamines excretion patterns in pig and raturine by ion-exchange liquid chromatography with electrochemical detection. J Chromatogr B Biomed Sci Appl. 1997b; 703:15–23. 9448058 10.1016/s0378-4347(97)00390-3

[26] Levine TR, Hullett CR. Eta squared, partial eta squared, and misreporting of effect size in communication research. Hum Commun Res. 2006; 28:612–625.

[27] Batterham AM, Hopkins WG. Making meaningful inferences about magnitudes. Int J Sports Physiol Perform. 2006; 1:50–57. 19114737

[28] Coutts A, Reaburn P, Piva TJ, Murphy A. Changes in selected biochemical, muscular strength, power, and endurance measures during deliberate overreaching and tapering in rugby league players. Int J Sports Med. 2007a; 28:116–124. 16835824 10.1055/s-2006-924145

[29] Coutts AJ, Reaburn P, Piva TJ, Rowsell GJ. Monitoring for overreaching in rugby league players. Eur J Appl Physiol. 2007b; 99:313–324. 17219174 10.1007/s00421-006-0345-z

[30] Kraemer WJ, French DN, Paxton NJ, Häkkinen K, Volek JS, Sebastianelli WJ, et al. Changes in exercise performance and hormonal concentrations over a big ten soccer season in starters and nonstarters. J Strength Cond Res. 2004; 18:121–128. 14971972 10.1519/1533-4287(2004)018<0121:ciepah>2.0.co;2

[31] Bright GM. Corticosteroid-binding globulin influences kinetic parameters of plasma cortisol transport and clearance. J Clin Endocrinol Metab. 1995; 80:770–775. 7883829 10.1210/jcem.80.3.7883829

[32] Esler M, Jennings G, Korner P, Willet I, Dudley F, Hasking G, et al. Assessment of human sympathetic nervous system activity from measurements of norepinephrine turnover. Hypertension. 1988; 11:3–20. 2828236 10.1161/01.hyp.11.1.3

[33] Iyer EM, Banerjee PK, Sengupta AK, Baboo NS. Neuroendocrine responses of flight cadets during midterm tests and of fighter pilots during tail chase sorties. Aviat Space Environ Med. 1994; 65:232–336. 8185553

[34] Issurin VB. New horizons for the methodology and physiology of training periodization. Sports Med. 2010; 40:189–206. 20199119 10.2165/11319770-000000000-00000

[35] Bosquet L, Montpetit J, Arvisais D, Mujika I. Effects of tapering on performance: a meta-analysis. Med Sci Sports Exerc. 2007; 39:1358–1365. 17762369 10.1249/mss.0b013e31806010e0

[36] Marrier B, Robineau J, Piscione J, Lacome M, Peeters A, Hausswirth C, et al. Supercompensation kinetics of physical qualities during a taper in team-sport athletes. Int J Sports Physiol Perform. 2017; 12:1163–1169. 28121198 10.1123/ijspp.2016-0607

